# Predominance of asymptomatic and sub-microscopic infections characterizes the *Plasmodium* gametocyte reservoir in the Peruvian Amazon

**DOI:** 10.1371/journal.pntd.0005674

**Published:** 2017-07-03

**Authors:** Eduard Rovira-Vallbona, Juan José Contreras-Mancilla, Roberson Ramirez, Mitchel Guzmán-Guzmán, Gabriel Carrasco-Escobar, Alejandro Llanos-Cuentas, Joseph M. Vinetz, Dionicia Gamboa, Anna Rosanas-Urgell

**Affiliations:** 1 Department of Biomedical Sciences, Institute of Tropical Medicine, Antwerp, Belgium; 2 Instituto de Medicina Tropical Alexander von Humboldt, Universidad Peruana Cayetano Heredia, Lima, Peru; 3 Laboratorio Satelite Iquitos UPCH-UCSD, Universidad Peruana Cayetano Heredia, Loreto, Peru; 4 Department of Medicine, University of California San Diego, San Diego, California, United States of America; 5 Departamento de Ciencias Celulares y Moleculares, Facultad de Ciencias y Filosofia, Universidad Peruana Cayetano Heredia, Lima, Peru; Johns Hopkins Bloomberg School of Public Health, UNITED STATES

## Abstract

Malaria transmission requires that *Anopheles* mosquitoes ingest *Plasmodium* gametocyte stages circulating in the human bloodstream. In the context of malaria elimination, understanding the epidemiology of gametocytes relative to all *Plasmodium* infections and the contribution of asymptomatic and sub-microscopic parasite carriers to the gametocyte reservoir is necessary, especially in low endemic settings with predominance of *P*.*vivax*. A 13-month longitudinal study was conducted in two communities (n = 1935 individuals) of Loreto Department, Peru, with five active screenings for *Plasmodium* infections and gametocyte stages by quantitative real-time PCR (qPCR) and reverse transcription (RT)-qPCR, respectively. Parasite prevalence by qPCR was 7.2% for *P*.*vivax* (n = 520/7235; range by survey 6.0%-8.1%) and 3.2% for *P*.*falciparum* (n = 235/7235; range by survey 0.4%-7.7%). Sub-microscopic infections accounted for 73.5% of *P*.*vivax* (range by survey 60%-89%) and almost the totality of *P*.*falciparum* cases. Gametocytes were found in 28.4% *P*.*vivax* infections (range by survey 18.7%-34.1%), with a peak of 61.5% in one community at the start of the transmission season. About 59.8% of all *P*.*vivax* gametocyte carriers were asymptomatic and 31.9% were sub-microscopic. Age patterns for gametocyte prevalence paralleled asexual stage infections and peaked among >15–25 year old individuals. Asexual parasite density was found to be the strongest predictor for *P*.*vivax* gametocyte presence in longitudinal multivariate analysis (odds ratio 2.33 [95% confidence interval 1.96, 2.78]; *P*<0.001). Despite significant differences in seasonality patterns and *P*.*vivax* prevalence found at the local scale, sub-microscopic and asymptomatic infections predominate and contribute significantly to the gametocyte reservoir in different communities of the Peruvian Amazon. Control and elimination campaigns need sensitive tools to detect all infections that escape routine malaria surveillance, which may contribute to maintain transmission in the region.

## Author summary

Malaria elimination, *i*.*e*. the complete interruption of parasite transmission in a region, is in the agenda of health authorities in countries that achieved substantial reduction of the disease burden in the past decade. However, our understanding of transmission epidemiology for low transmission areas where *Plasmodium vivax* is endemic, like the Amazon basin, is still limited. In this study, we describe the prevalence and risk factors for carrying the parasite stages that are transmitted to the mosquito vectors, named gametocytes, in 1935 individuals from two communities of the Peruvian Amazon that were regularly screened during 1 year. We report that malaria infections with no clinical symptoms and those with parasite levels below microscopy detection threshold, account for two thirds of all *P*.*vivax* infections with gametocytes, and that the highest infection rate is found among young adults. In addition, almost the totality of *P*.*falciparum* infections detected was sub-microscopic. Because all these infections escape current malaria surveillance systems -based on passive case detection and/or microscopy diagnosis-, new approaches are necessary to target all infections in order to eliminate the malaria transmission reservoir in Peru.

## Introduction

As countries in the Americas develop plans for malaria pre-elimination, a better understanding of *Plasmodium* transmission epidemiology is necessary to implement effective interventions, in particular for *Plasmodium vivax* [1,2]. In Peru, similar to other regions in Amazonia, *P*. *vivax* is responsible for about 80% of all malaria infections [3], with asymptomatic low-grade parasitemia reported for ≈75% of them [4,5]. Between 2005 and 2011, malaria was reduced by over 50% thanks to intensification of control efforts and the PAMAFRO campaign (*Malaria Control Program in Border Areas of the Andean Region*), which included active screening and treatment and distribution of insecticide-treated nets [6]. Unfortunately, and coinciding with a reduction of control measures, a steady increase in cases has been reported in the country since 2012 characterized by high levels of transmission heterogeneity at the local scale [6,7]. In this context, accurate description of the human malaria reservoir and the contribution of asymptomatic and sub-microscopic infections to transmission is lacking.

Malaria transmission from the human host to the mosquito vector requires that *Anopheles* species ingest mature sexual forms of *Plasmodium* parasites, named gametocytes, during a blood meal [8]. Therefore, gametocyte carriage can be used as indirect estimator of the infectiousness potential of individuals in molecular epidemiology studies (taking into account that mosquito infections may be modulated by inter-individual variation in yet poorly understood vector, parasite and host factors [8,9]). The characterization of the gametocyte reservoir includes the detection of sexual stage infections, their quantification relative to asexual parasites, the identification of factors that determine gametocyte emergence, determining how gametocyte carriage changes over time, or what is the spatial clustering of sexual stage infections.

Most of the current knowledge on *Plasmodium* gametocyte epidemiology comes from studies on *Plasmodium falciparum* species. Sexual *P*.*falciparum* stages are normally observed in peripheral blood ≈7–12 days after initial asexual infection is established. A recent clinical trials meta-analysis showed that, on average, 12.1% of infections carry gametocytes at enrolment by light microscopy (LM), and that these infections are associated with anaemia, absence of fever and low asexual parasite densities; after treatment, gametocyte rate is higher among individuals taking sulphadoxine pyrimethamine or chloroquine as compared to artemisinin combination therapies (ACT) [10]. Community-based cross-sectional and cohort studies, which take into account asymptomatic infections, have set the proportion of *P*.*falciparum* gametocyte carriers in a wide range from ≈10% by LM in Senegal to as high as 61% in Papua New Guinea (PNG) by reverse-transcription quantitative PCR (RT-qPCR) or 70.1% in Burkina Faso by QT-NASBA [4,11–16]. These studies showed that gametocyte carriage is higher in young age groups [11–15], and correlates with asexual parasite densities -when measured by molecular methods [11,15]-, presence of fever or clinical malaria [13–15], haemoglobin C and S variants [16,17], and blood groups O and B [14]. On the other hand, *P*.*vivax* transmission epidemiology and the characteristics of its infectious reservoir have been less studied [8]. *P*.*vivax* gametocytes are produced shortly after initial asexual wave of parasitemia in the bloodstream, have a shorter lifetime, and are suggested to infect mosquitoes more efficiently than *P*.*falciparum* [8,18]. In recent community based surveys, the proportion of infections with *P*.*vivax* sexual stages was found to reach >50% by LM [4,19], whereas by RT-qPCR 23.5%-96% of infections carried gametocytes in studies in Oceania and Brazil [15,20,21]. Like for *P*.*falciparum*, young age [15,20,22] and high asexual parasite density were described as the main predictors [15,19,21–25]. Low haemoglobin levels and fever have also been linked with *P*.*vivax* gametocyte presence [15,23], although equally high rates of gametocytes were reported in symptomatic and asymptomatic infections in Brazil [21,26].

Here, we aim to improve our understanding of *Plasmodium* gametocyte epidemiology and generate region specific data that can inform health authorities to tailor transmission-blocking strategies to the local context of transmission heterogeneity. A longitudinal cohort study was conducted in two separate communities, where all individuals were regularly screened for asexual and sexual stage infections using molecular tools for one year. The factors that contribute to gametocyte carriage, with a focus on asymptomatic and sub-microscopic carriers, are discussed.

## Methods

### Study area

**Fig 1:**
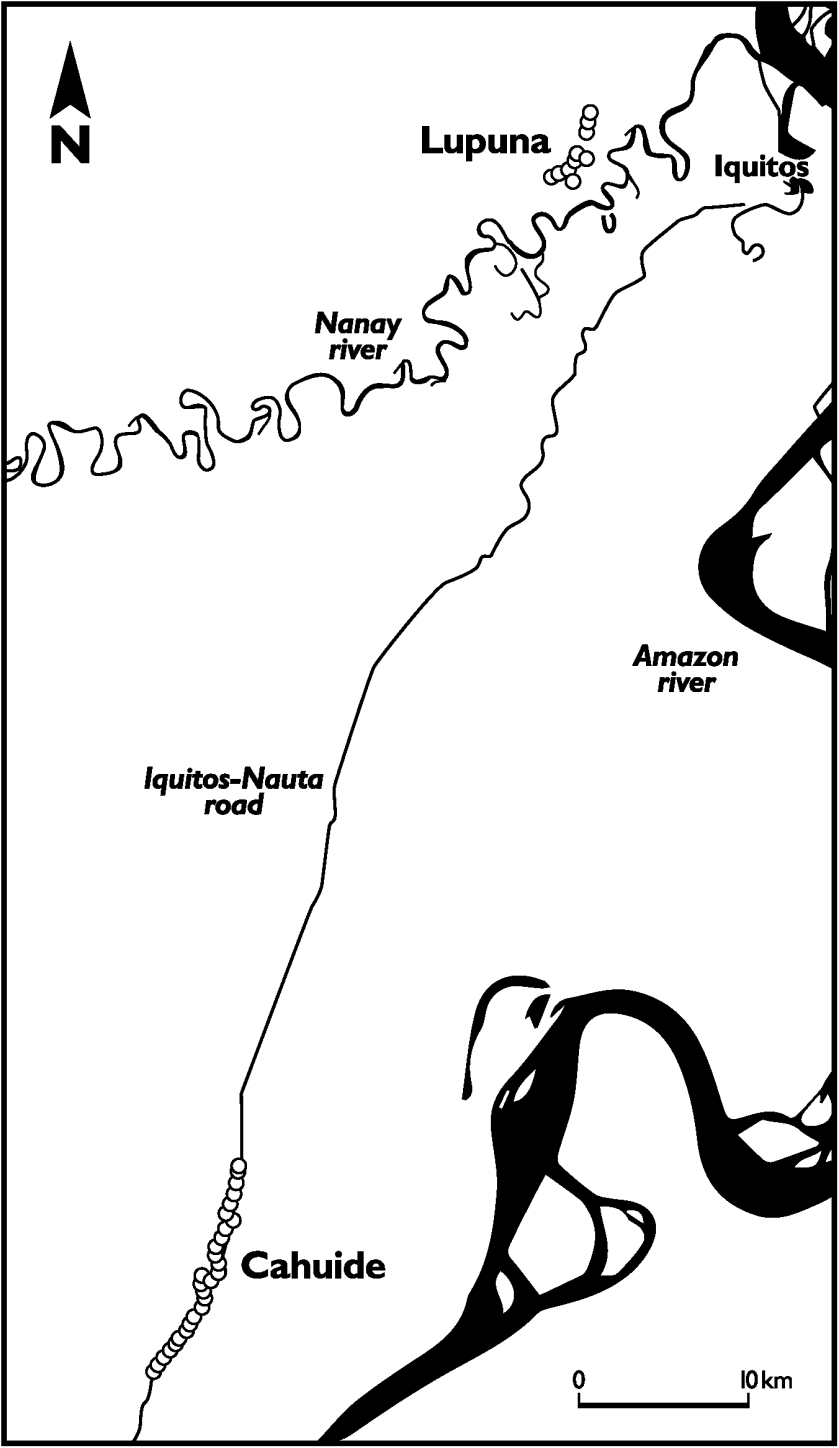
Map of the study area. Location of Cahuide and Lupuna communities relative to Iquitos city, the Amazon and Nanay rivers and Iquitos-Nauta road.

The study was conducted in the communities of Cahuide and Lupuna, Maynas Province, Loreto Department, Peru (Fig 1). The region is characterized by a tropical climate with a mean temperature of 27.5°C. Despite significant transmission heterogeneity, overall malaria is perennial with marked seasonality and a peak in April-June. In 2013, Loreto Department accounted for 89.5% (43284) of all malaria cases in Peru, being 81.9% (35458) caused by *P*.*vivax* [27]. Cahuide is a rural community 57 km away from Iquitos city. Houses are scattered along 9 km of the Nauta road, at the intersection with Itaya river. The area is characterized by road-driven deforestation and palm-roof production. Monthly entomological inoculation rate (EIR) range was 0–2.52 infective bites/person/month in 2012 [28]. On the other hand, Lupuna community includes three villages located 500–1000m away from the left bank of river Nanay (Fig 1). Although closer to Iquitos city than Cahuide, the area is only accessible by boat and mainly forested. Population is relatively stable and works in agricultural activities. Monthly EIR range estimates for Lupuna were 0–1.98 in 2012. In both sites *Anopheles darlingi* is the dominant vector species [28]. Malaria control in the area relies mainly on passive case detection and LM diagnosis performed at health posts. Active case detection campaigns by LM are performed only when outbreaks or unusual rises in case numbers are reported in a community.

### Study design and sample collection

As part of a 3-year prospective population-based cohort study with longitudinal follow-up, we analyzed gametocyte carriage every three months from December 2013 to December 2014 (five surveys). A total of 1935 individuals from 442 households aged >3 months were included. At each survey, all individuals were screened for malaria symptoms and completed a questionnaire with demographic data. Blood smears, filter paper blood spots, and three blood drops (≈50 μl ±10 μl) into 250 μl of RNAprotect stabilizer reagent (Qiagen), were collected from finger pricks. RNAprotect-blood mixture was kept cold and transferred to Iquitos for storage at -20°C. Blood smears were stained with Giemsa and examined for malaria parasites under 700x magnification. Parasite counts per 200 leukocytes were used to estimate parasite density, assuming 8000 leukocytes in 1 μl of blood. All infections diagnosed by LM were treated according to National Guidelines from Peruvian Ministry of Health (MINSA), regardless of symptoms.

### Total parasite quantification by qPCR

DNA was extracted from one punch of dried blood spots on filter paper (≈25mm^2^, ≈7.5 μl) using QIAamp DNA Mini kit (Qiagen) following manufacturer’s instructions, and eluted in 150 μl of AE buffer. Identification and quantification of *Plasmodium* species was done by real-time qPCR protocol targeting *18S* ribosomal genes, adapted from Mangold *et al* [29]. Briefly, 5 μl of DNA were added to a final reaction volume of 25 μl including 12.5 μl of PerfeCTa SYBR Green FastMix (Quantabio) and 300nM primers PL1473F18 and PL1679R18 (IDT), and run in a CFX Connect thermocycler (Bio-Rad). Species were identified by melting temperature (T_m_) curve analysis using CFX Manager software (Bio-Rad), with a T_m_ of 77°C (±1°C) for *P*.*vivax* and 73°C (±1°C) for *P*.*falciparum*. Plasmids with species-specific *18S* gene inserts were used as controls. The limit of detection was set at the dilution where at least 60% of the replicates were positive, and corresponded to 1 copy/reaction for both species (*i*.*e*. 4 copies/μl of blood in study samples). Sample quantification was done using standard curves built from 1:10 plasmid dilution series. Only samples with both valid Ct and T_m_ values were considered positive.

### Gametocyte quantification by RTqPCR

The presence of mature gametocytes in qPCR positive samples was determined by one-step reverse transcription qPCR (RTqPCR), targeting *Pfs25* (*P*.*falciparum*, PF3D7_1031000) and *Pvs25* (*P*.*vivax*, PVX_111175) mature gametocyte specific gene transcripts. RNA was extracted from RNAprotect samples using RNeasy Mini Kit columns (Qiagen) and eluted in 50 μl of THE-RNA Storage Solution (Ambion). Contaminant genomic DNA was removed by treatment with TURBO DNAse (Ambion) for 1h at 37°C. Parasite RNA presence was confirmed in a random set of 10% of the samples using RT (Maxima First Strand cDNA Kit, Thermo) and *18S* qPCR. Gametocytes RTqPCR was performed in a LightCycler 480 using LightCycler Multiplex RNA Virus Master kit (Roche), with primers and HEX(*P*.*falciparum*)- and FAM(*P*.*vivax*)-labelled hydrolysis probes from Wampfler *et al* [30] (IDT). All samples were tested in duplicate reaction and controls without RT enzyme were added to exclude false positives due to the presence of genomic DNA. Analysis was done in LightCycler480 software version 1.5.0. Replicates with Ct difference >1 were repeated. *P*.*falciparum* gametocyte densities were quantified using a standard curve generated from *in vitro* cultured gametocytes. Briefly, *P*.*falciparum* 3D7-E5 strain (kindly provided by Dr. Alfred Cortés, ISGlobal, Barcelona) was synchronized with 5% sorbitol and induced for gametocytogenesis by stress with partially spent medium for 2 consecutive days. Asexual stages were removed by 50 mM N-acetyl-glucosamine treatment until day ≈12–14, when mature stage V gametocytes were harvested and concentrated using MACS magnetic separation (LD columns, Milteny Biotec). A 7-point 1:10 dilution series ranging from 100.000 to 0.1 gametocytes/μl was prepared in whole blood and resuspended in RNAprotect as described above for gametocyte density quantification. Due to the lack of *P*.*vivax in vitro* culture, *P*.*vivax* densities were first estimated from a *P*.*falciparum* standard curve quantified using a FAM-labelled *Pfs25* probe, and a correction factor for the differential expression of *Pvs25 vs Pfs25* was then applied using recently published *P*.*falciparum* and *P*.*vivax* gametocyte trend lines [15].

### Definitions and statistical methods

Clinical malaria was defined as confirmed *Plasmodium* infection by microscopy and/or qPCR presenting with fever, chills and/or headache at the time of visit, or reporting one of these symptoms during the previous 7 days. Asymptomatic individuals were defined as those with confirmed infection not presenting any of these symptoms at the time of visit or in the past 7 days. Prevalence was defined as the number of parasite carriers out of the total population, whereas the term 'rate' was used to define the proportion of asymptomatic/sub-microcopic/gametocytemic individuals out of the total number of infections. Because only malaria positive samples were processed for RNA-based detection of gametocytes, gametocyte population prevalence is considered an estimate. Incidence of gametocyte carriage was calculated as the number of new sexual stage infections per 1000 person/year. Time-at-risk was calculated as 90 days per each survey in which an individual participated after study initiation. When treatment was reported, 15 days were subtracted as a risk-free time [31]. For individuals positive for gametocytes in two consecutive surveys without reported treatment, only first observation was counted, whereas those positive in two non-consecutive surveys were considered as independent infections. For parasite densities, only results by molecular methods are reported.

Comparisons between demographic categorical variables were done using chi-square or Fisher’s exact test, and age means were compared by t-test. Non-parametric Kruskal-Wallis test was used to compare parasite densities. Multilevel regression models were used to determine risk factors for *Plasmodium* infection during longitudinal follow-up in Stata software (version 11.0; StataCorp), with each observation nested by individual and individuals grouped by household. Data were set as panel ordered by time of screening. Logistic regression was used for association analysis with parasite prevalence and gametocyte rate, while log transformed parasite densities were analyzed by linear regression. Univariate models were first run including as independent variables demographic data (age, gender, pregnancy status, community of residence, bed net coverage), work status and occupation, house construction materials, clinical data (malaria symptoms, history of malaria in previous year), and 18S copy numbers–the latter for gametocyte associations only-. Multivariate models were built with stepwise forward selection of all co-variables with 5% significance level in univariate analysis plus age. Predictors added in decreasing order of significance were kept if its addition led to a decrease in Akaike Information Criterion value. Overall significance for variables with multiple categories was estimated using Wald test. *P*-values <0.05 were considered statistically significant. Data were plotted using Prism 7 (GraphPad).

### Spatial statistics

Spatial scan analysis was conducted to identify purely spatial (by survey) and spatio-temporal (all surveys) clusters of gametocyte carriers at households or village level. Separate analysis were run for each community among all qPCR-positive individuals in SaTScan 9.4.2 [32]. Briefly, circular windows of multiple sizes containing a maximum of 30% of the population were applied, in which the probability that the observed prevalence is higher than the expected under the hypothesis of no clustering was tested using a Bernoulli distribution model. *P*-values were computed across 999 Monte-Carlo replications. Because spatial scan statistics may have limitations to identify hotspots in areas where data is distributed linearly like Cahuide [33], autocorrelation analysis was performed in ArcMap 10.4 (ArcGIS, ESRI, [34]) using Getis-Ord Gi* statistic and False Discovery Rate correction for multiple testing. Distance bands were calculated using Incremental Spatial Autocorrelation tool in windows of 25 m and the distance corresponding to first Z-score peak was selected (200m). Results were mapped in QGIS 2.12.

### Ethics statement

Written informed consent was obtained from all individuals -or their parents or guardians in the case of minors- before conducting any study activity. The study received ethical approval from the *Comité Institucional de Ética*, Universidad Peruana Cayetano Heredia (Lima, Peru; code SIDISI 57395), and the Institutional Review Board, Institute of Tropical Medicine (Antwerp, Belgium; reference 1080/16).

## Results

### Characteristics of study population at screening

Out of 1935 censed individuals, 1369 (71%) participated in ≥4 surveys, 290 (15%) participated in 3 surveys and 276 (14%) in ≤2 surveys (Table 1 and S1 Table). 7265 samples were collected over the study period, with full clinical data records available for 5746 visits. Individuals from Cahuide were younger and reported a significantly higher number of malaria cases in the year prior to study initiation than individuals from Lupuna (Table 1, *P*<0.001). However, symptomatic malaria by LM was more frequent in Lupuna during the time of study (*P*<0.001). About 96% of households (425/442) reported having bed nets, either long-lasting insecticide-treated or locally produced using *tocuyo* textile. Recruitment was significantly lower in March compared to other months (S1 Table). Overall, individuals sampled in this particular survey did not differ in age, gender, occupation or type of housing, but had significantly higher malaria history in the previous year compared to other surveys (*P* = 0.001). The difference in malaria history between surveys was not observed after stratifying by community (*P*>0.852).

**Table 1:** Characteristics of the study cohort at screening (December 2013- December 2014).

	Total		Cahuide		Lupuna		
	n	%	n	%	n	%	*P*-value
Individuals	1935	**-**	1062	**-**	873	**-**	*NA*
Age, *mean (95% CI)*	27.8 (4, 67)		26.0 (4, 65)		30.2 (5, 70)		**<0.001**
Age							
≤5y	202	10.4	123	11.6	79	9.0	**<0.001**
>5y - 10y	286	14.8	167	15.7	119	13.6	
>10y - 15y	250	12.9	161	15.2	89	10.2	
>15y - 25y	311	16.1	153	14.4	158	18.1	
>25y	886	45.8	458	43.1	428	49.0	
Male/female ratio	1.1	-	1.1	-	1.0	-	0.555
Work status							
Employed	658	34.0	348	32.8	310	35.5	
Students or children	854	44.1	497	46.8	357	40.9	
Other (including housework)	423	21.9	217	20.4	206	23.6	**0.034**
Households	442	-	241	-	201	-	*NA*
House wall materials							**<0.001**
Brick, cement	39	8.8	10	4.2	29	14.4	
Wood	344	77.8	200	83.0	144	71.6	
Palm	38	8.6	16	6.6	22	11.0	
Other	21	4.8	15	6.2	6	3.0	
Had malaria previous year	599	31.3	474	44.8	125	14.6	**<0.001**
Number of visits*^a^*	7265	-	4091	-	3174	-	*NA*
Visits with symptoms*^a^*^,^*^b^*	762	13.3	284	9.5	478	17.3	**<0.001**
Clinical malaria cases*^a^*^,^*^c^*							
*P*. *vivax*	56	1.0	17	0.6	39	1.4	0.734
*P*. *falciparum*	2	0.03	0	0	2	0.1	**-**

^a^total for 5 surveys

^b^fever, headache and/or chills (among those with full clinical data available at the time of visit and during the previous 7 days)

^c^by light microscopy at screening. *NA*, not applicable; *CI*, confidence interval.

### *Plasmodium* infections (all stages)

**Fig 2:**
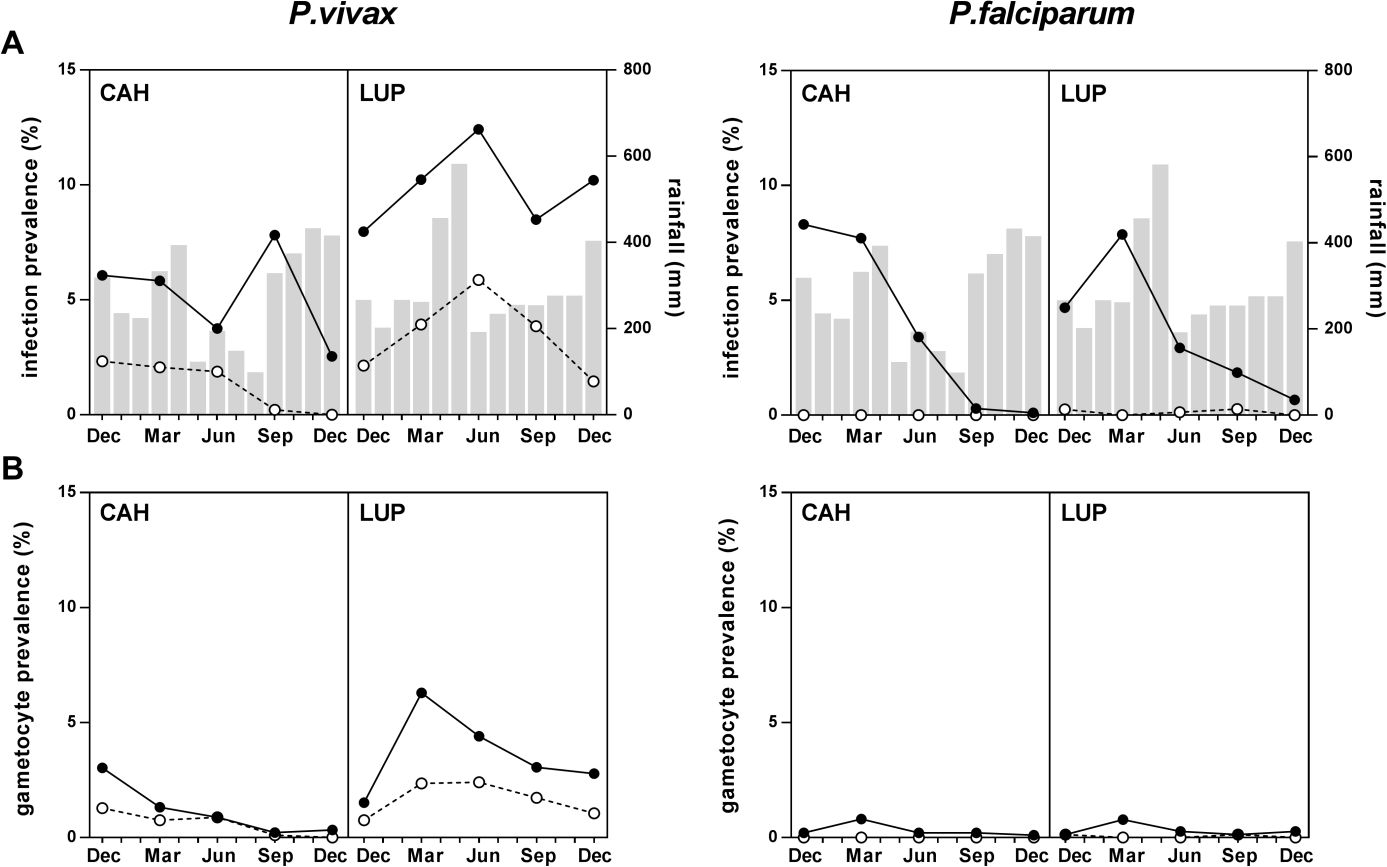
*Plasmodium* infections and gametocyte carriage from December 2013 to December 2014. A) *P*.*vivax* and *P*.*falciparum* prevalence by LM (white circles) and qPCR (black circles) in Cahuide (CAH) and Lupuna (LUP). Monthly rainfall records are shown in bars, corresponding to station 153-Morallillo (for CAH) and station 154-Iquitos (for LUP; source: http://www.senamhi.gob.pe/ [35]). B) *P*.*vivax* and *P*.*falciparum* gametocyte prevalence by LM (white circles) and RTqPCR (black circles).

**Fig 3:**
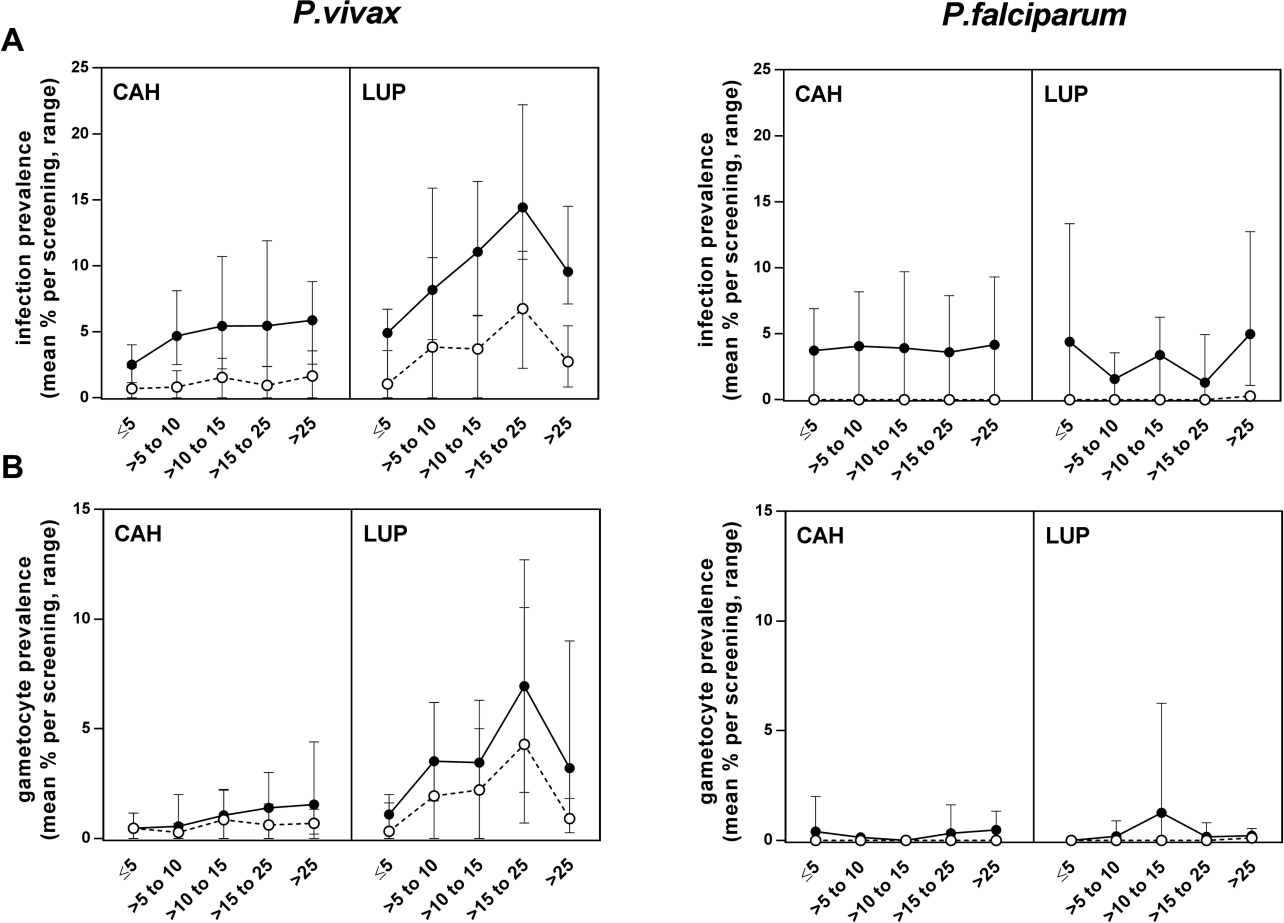
*Plasmodium* infections and gametocyte carriage by age. A) *P*.*vivax and P*.*falciparum* prevalence by LM (white circles) or qPCR (black circles) in Cahuide (CAH) and Lupuna (LUP). B) Gametocyte prevalence by LM (white circles) or RTqPCR (black circles). Symbols correspond to the mean of all surveys and error bars indicate minimal and maximal prevalence observed during surveys.

*P*.*vivax* was the most common malaria species both by LM (156/7265, 2.1% [range by survey 0.7%-3.7%]) and qPCR (520/7265, 7.2% [6.0%-8.1%]). A total of 432 individuals (22.3% of the population) had a *P*.*vivax* infection at some point during follow-up, and 71 (3.7%) were positive in more than one survey. By qPCR, prevalence was higher in Lupuna (mean 9.9% [range by survey 8%-12.4%]) than Cahuide (5.2% [3.8%-7.8%], *P*<0.001, Fig 2A), with a marked seasonal pattern peaking in June after major rainfall. On the contrary, Cahuide showed an overall decrease in infections despite a peak in September, with no positive blood smears by the end of the follow-up period in December 2014. *P*.*vivax* infections increased with age until 25 years old (Fig 3A), and peaked among >15–25 year old in Lupuna (82/545 by qPCR, 15% [range by survey 10.5%-22%], *P<*0.001; Fig 3A). Overall median *P*.*vivax* density was 112 copies of *18S*/μl (inter-quartile range [IQR] 44, 536), with higher parasite densities in Lupuna (140 copies/μl [IQR 56, 1016]) than Cahuide (80 copies/μl [IQR 32, 296], *P*<0.001; S1 Fig).

*P*.*falciparum* parasites were detected in 5/7265 blood smears and 235/7265 qPCR samples (3.2% [range by survey 0.4–7.7%]), originating from 222 different individuals (11.5% of the population). *P*.*falciparum* qPCR infections were more frequent in Cahuide (3.6% [range by survey 0.1%-8%]) than Lupuna (2.8% [0.7–4.7%], *P*<0.001; Fig 2A), and decreased with time down to an overall prevalence of 0.4% (6/1657) in the last survey. No differences in *P*.*falciparum* prevalence were found by age groups in any community (*P*>0.174, Fig 3A). Median *P*.*falciparum* density (28 copies/μl [IQR 12, 56]; S1 Fig) was lower than for *P*.*vivax* (*P*<0.001).

High rates of sub-microscopic and asymptomatic infections were found throughout the study period for both species (Table 2). Sub-microscopic infections accounted for 73% (range by survey 60%-89%) of all *P*.*vivax* and 97.8% (88%-100%) of all *P*.*falciparum* positive samples. Using the qPCR result for clinical malaria case definition, the number of *P*.*vivax* clinical cases increased from 56 (by LM at screening) to 101, and the number of *P*.*falciparum* clinical cases from 2 to 30. Still, the large majority of infections detected after qPCR were asymptomatic (77.2% [range by survey 72%-82%] for *P*.*vivax* and 82.7% [60%-90%] for *P*.*falciparum*). Parasite densities in *P*.*vivax* asymptomatic carriers were lower (92 copies/μl [IQR 40, 292]) than those in clinical cases (544 copies/μl [IQR 88, 5712], *P*<0.001). No difference in parasite densities was found between *P*.*falciparum* asymptomatic and symptomatic infections (7 copies/μl [IQR 3, 14]) vs 5 copies/μl [IQR 2, 13], *P* = 0.364). By community, Cahuide showed significantly higher rates of sub-microscopic infections for both species (*P<*0.005), as well as higher asymptomatic rates for *P*.*falciparum* (*P* = 0.015; Table 2).

**Table 2:** Sub-microscopic and asymptomatic *Plasmodium* infection rates, by qPCR/RTqPCR.

	Total		Cahuide		Lupuna		
	n/N	% (range)^a^	n/N	% (range)^a^	n/N	% (range)^a^	*P*-value
***P*.*vivax* infections**							
Sub-microscopic rate	380 / 520	73 (60–89)	172 / 210	82 (65–100)	208 / 310	67 (55–86)	**0.005**
Sub-microscopic+gametocytemic rate	76 / 520	15 (9–20)	26 / 210	12 (3–33)	50 / 310	16 (10–39)	0.235
Asymptomatic rate^b^	342 / 443	77 (72–82)	139 / 170	82 (47–94)	203 / 273	74 (65–87)	0.071
Asymptomatic+gametocytemic rate^b^	73 / 443	16 (9–21)	18 / 170	11 (2–24)	55 / 273	20 (17–42)	**0.008**
***P*.*falciparum* infections**							
Sub-microscopic rate	230 / 235	98 (88–100)	147 / 147	100 (100–100)	83 / 88	94 (86–100)	**0.003**
Sub-microscopic+gametocytemic rate	16 / 235	7 (2–50)	11 / 147	7 (3–100)	5 / 88	6 (0–40)	0.060
Asymptomatic rate^b^	143 / 173	83 (60–90)	87 / 98	89 (84–100)	56 / 75	75 (50–89)	**0.015**
Asymptomatic+gametocytemic rate^b^	12 / 173	7 (0–40)	8 / 98	8 (0–100)	4 / 75	5 (0–25)	0.468

^a^ range by survey

^b^among those with full clinical data available at the time of visit and during the previous 7 days.

### Gametocyte carriage

*P*.*vivax* gametocytes were observed in 72 blood smears (1% population prevalence; Fig 2B). By molecular methods, gametocytes were detected in 143 samples (2% estimated population prevalence) originating from 135 different individuals (7%); only 8/135 (6%) individuals carried gametocytes in more than one time-point. The estimated annual incidence for *P*.*vivax* gametocyte carriage was 72 sexual-stage infections/1000 person-year (136.2 for Lupuna and 72 for Cahuide). Seasonal trends in gametocyte prevalence differed by community: in Lupuna, gametocytes by RTqPCR peaked in March (8/127, 6.3% est. population prevalence; Fig 2B) and decreased afterwards (21/74, 2.8% est. population prevalence in December), whereas in Cahuide gametocyte prevalence decreased during the whole study period, as did asexual parasitemia (Fig 2B). Age patterns for gametocyte prevalence were similar than those for asexual stage infections, and highest among the >15–25 year old group (Fig 3B). *P*.*vivax* gametocyte densities by RTqPCR did not vary by survey (*P* = 0.261) or community (*P* = 0.319; S1 Fig). *P*.*falciparum* gametocytes were detected in 18 samples by RTqPCR (0.3% population prevalence). Annual incidence was 10.7 cases/1000person-year (11.1 in Cahuide and 10.1 in Lupuna).

**Fig 4:**
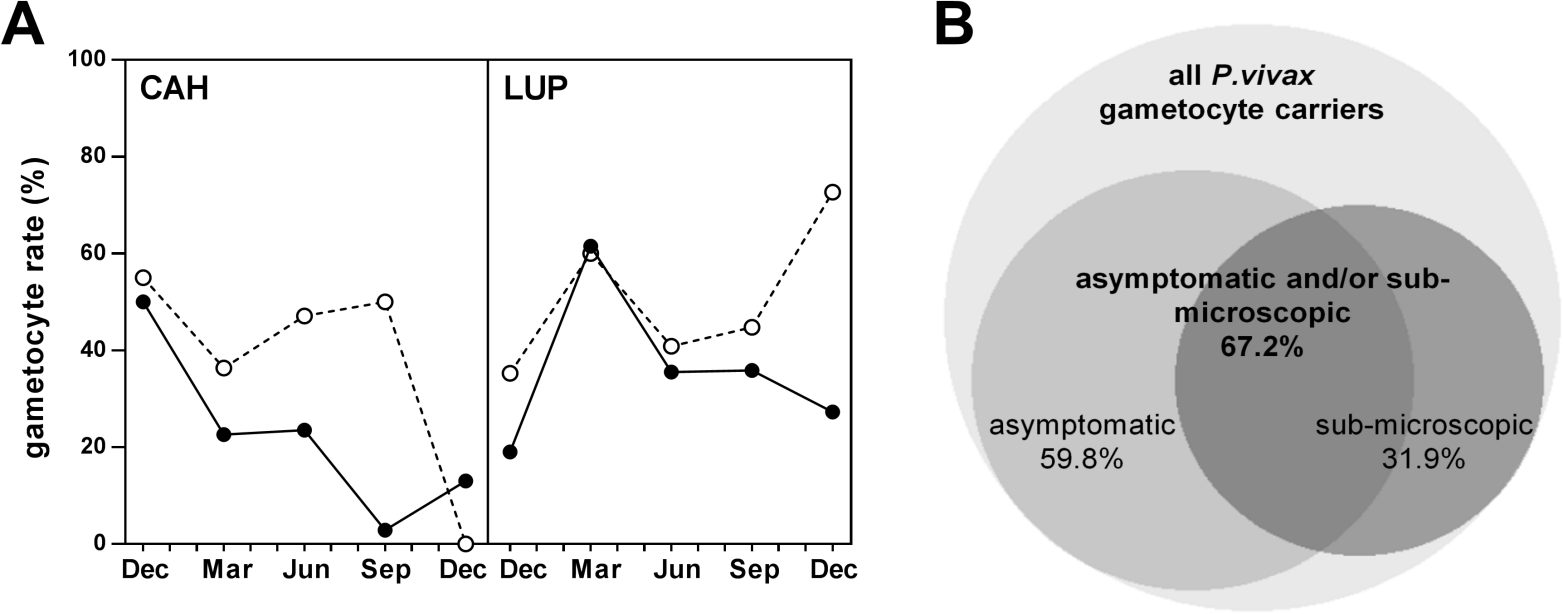
Proportion of *P*.*vivax* infections carrying gametocytes. A) *P*.*vivax* gametocyte rate among all infected individuals (by LM in white circles, or by qPCR/RTqPCR in black circles). B) Venn diagram for the contribution of asymptomatic and sub-microscopic infections to total *P*.*vivax* gametocyte reservoir, by qPCR/RTqPCR (built using BioVenn [36]; only samples with complete clinical records were considered).

Among positive samples, the proportion of *P*.*vivax* infections carrying gametocytes was 46.2% (72/156 [range by survey 42.6%-72.7%]) for blood smears and 28.4% (143/520; 18.7%-34.1%) by RTqPCR (Fig 4A). *P*.*vivax* gametocyte rates were higher in Lupuna (35.9% [range by survey 19%-61.5%]) than Cahuide (22.4% [3%-50%], *P* = 0.001; Fig 4A) and did not vary significantly by age category (*P* = 0.191). The gametocyte rate for *P*.*falciparum* was 7.6% (18/235 [range by survey 3%-50%]) and was similar in both communities and in all age groups (*P*>0.234).

Sub-microscopic and/or asymptomatic infections with gametocytes were found to constitute a significant proportion of the total *P*.*vivax* reservoir, with rates ranging from 9% to 21% overall, and up to 42% for in Lupuna community (Table 2). If only gametocyte positive infections with full clinical data records are taken into account, sub-microscopic and/or asymptomatic infections represent 67.2% (82/122) of the gametocyte reservoir detected by molecular methods (Fig 4B). *P*.*vivax* gametocyte density in asymptomatic individuals (8.6 gametocytes/μl [IQR 1.9, 85]) did not differ from that in clinical cases (5.7 gametocytes/μl [IQR 2.0, 133.7], *P* = 0.696). On the other hand, the *P*.*falciparum* gametocyte reservoir was entirely constituted by infections that were either sub-microscopic and/or asymptomatic, although sample size was small (n = 18).

### Factors associated with *Plasmodium* infection and gametocyte carriage

Results for all co-variables associated with *P*.*vivax* infection in univariate analysis are provided as S2 Table, and were used to build multivariate models with stepwise forward selection. Final models showed that risk of *P*.*vivax* infection was highest in the >15–25 year old group, when compared to children under five (odds ratio, OR 3.1 [95% confidence interval (CI) 1.75, 5.49], *P*<0.001; Table 3). Other independent risk factors for *P*.*vivax* infection included residence in Lupuna, and living in houses built with palm or other materials -a category that includes mainly plastics and corrugated iron-, as compared to concrete or brick houses. Among infected individuals, total parasite density was the main independent predictor for the presence of gametocytes, with a 10% increase in *18S* copy numbers associated to double risk of carrying gametocytes (Table 3). In addition, a borderline association towards higher risk of gametocyte carriage was found for Lupuna. Age was not found to be a significant predictor for gametocyte risk in multivariate analysis (*P*>0.411).

**Table 3:** Multivariate models for *P*. *vivax* infection and gametocyte carriage.

	Parasite prevalence				Gametocyte rate			
Variable	OR	95% CI	*P*-value^a^		OR	95% CI	*P*-value^a^	
Age								
≤5y	1				1			
>5y - 10y	2.07	1.17, 3.69	**0.013**		0.88	0.16, 4.91	0.887	
>10y - 15y	2.64	1.47, 4.71	**0.001**		1.27	0.24, 6.87	0.780	
>15y - 25y	3.10	1.75, 5.49	**<0.001**		1.99	0.39, 10.24	0.411	
>25y	1.94	1.13, 3.32	**0.016**	*(****0*.*001****)*	1.58	0.32, 7.83	0.573	(*0*.*529*)
Village								
Cahuide	1				1			
Lupuna	1.88	1.45, 2.44	**<0.001**		1.87	1.01, 3.48	**0.050**	
House wall								
Brick, cement	1				-	-	-	
Wood	1.66	1.02, 2.70	**0.040**		-	-	-	
Palm	2.10	1.15, 3.84	**0.016**		-	-	-	
Other	3.31	1.62, 6.73	**0.001**	*(****0*.*007****)*	-	-	-	
*18S* copies/μl (log)	-	-	-		2.33	1.96, 2.78	**<0.001**	
Fever, headache or chills	2.09	1.59, 2.74	**<0.001**		-	-	-	

^a^result of Wald test shown in brackets. OR, odds ratio; CI, confidence interval.

Models for *P*.*vivax* parasite densities showed that *18S* copies were positively associated with clinical symptoms (OR 5.93 [95% CI 5.73, 9.17], *P*<0.001) as well as residence in Lupuna community (OR 1.59 [95% CI 1.05, 2.42], *P* = 0.028); S3 Table). Parasite density by *18S* was the only predictor of gametocyte density (OR 1.16 [95% CI 1.01, 1.32], *P* = 0.034; S3 Table), which did not differ between children and adults (*P>*0.188).

Association analysis was not attempted for *P*.*falciparum* sexual stage infections due to the low number of cases. Prevalence of *P*.*falciparum* asexual infection was associated with the absence of clinical symptoms, whereas no variables were associated with parasite density (S4 Table).

### Spatial clusters of *P*.*vivax* gametocyte carriage

**Fig 5:**
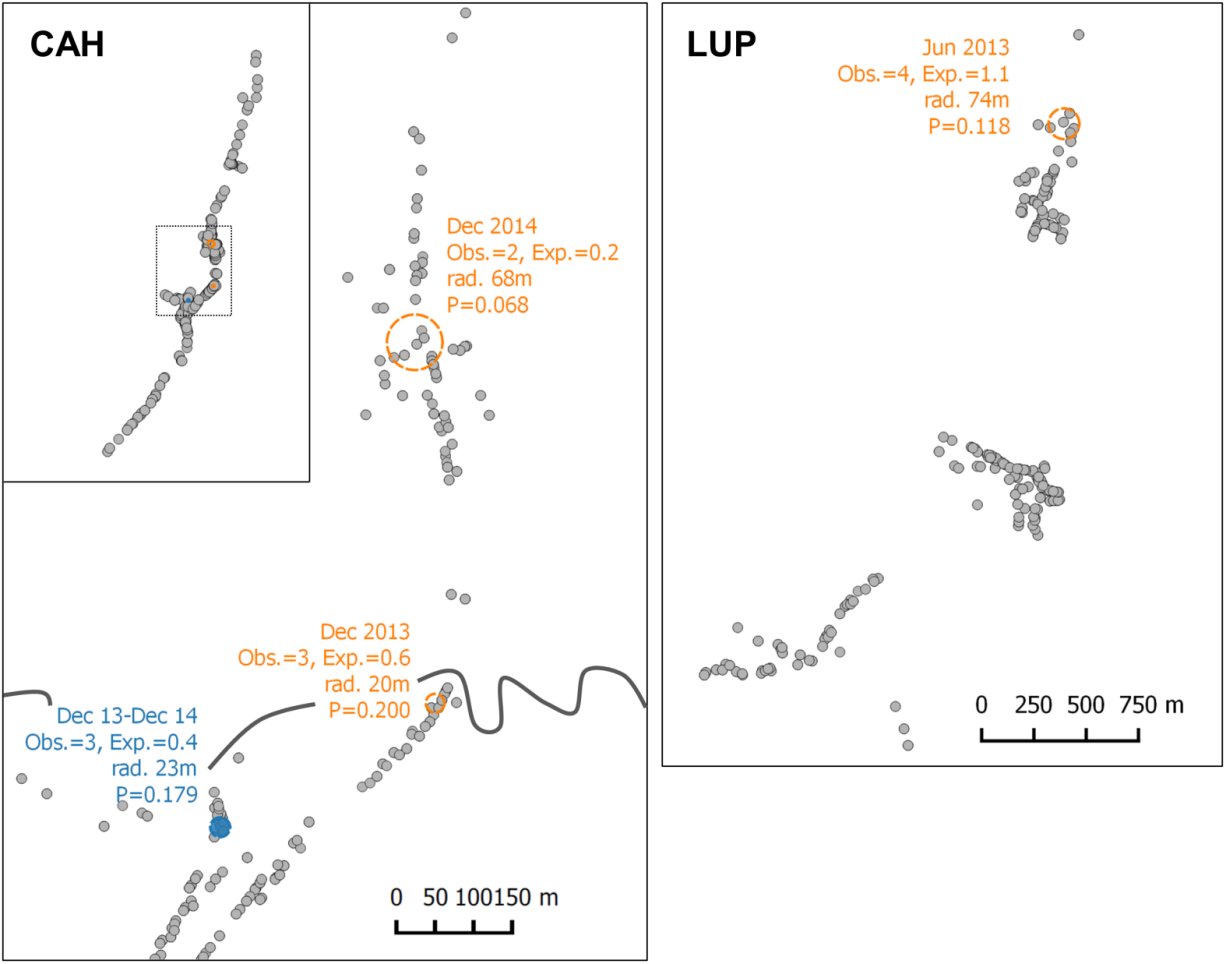
Spatial cluster analysis for *P*.*vivax* gametocyte carriage. Regions with higher-than-expected risk and *P*<0.200 are shown, based on SaTScan result. Orange circles indicate spatial clusters; blue circle indicates a spatio-temporal cluster.

SaTScan analysis could not identify spatial clusters with statistical significance (*P*<0.05), which may have been limited by the low number of observations per time-point together with uneven household geographical distribution. Four areas showed a higher-than-expected risk with significance thresholds below 20%, including three spatial clusters in Cahuide and Lupuna, and a small spatio-temporal cluster in Cahuide (Fig 5; relative risk range 3.8–21, *P*-value range 0.068–0.200). Individuals residing inside these areas did not differ in age, gender, type of household, occupation nor clinical characteristics compared to those outside the cluster (*P*>0.113). No hotspots at household level were identified by Getis-Ord Gi* statistic.

## Discussion

The data presented in this study constitutes the most accurate quantification of the *Plasmodium* gametocyte reservoir in Peru to date, and the largest conducted in the Amazon region. A previous study in Peru from 2003 using LM alone reported a gametocyte rate of 22% for *P*.*vivax* and 53% for *P*.*falciparum* [4], as compared to the 46% for *P*.*vivax* reported here by LM (almost all *P*.*falciparum* infections were sub-microscopic). This increase is in line with the overall reduction in endemicity in the country over the past decade, which may translate into a higher investment in sexual stages to sustain transmission [8]. On the other hand, the average gametocyte rate of 28.4% for *P*.*vivax* by RTqPCR compares to community-level data from a similar epidemiological context in Solomon islands (23.5% gametocyte rate in 2012) [20], but is lower than the 49% found in PNG in 2010 or a remarkably high 96% in Brazilian Amazon in 2011–12 [15,21]. Different factors may explain this variability. First, factors attributable to seasonality and study-designs. While the study in PNG was a cross-sectional conducted during the rainy-season, the study from Brazil estimated gametocyte rates from only a subset of surveys and samples (n = 55). Our data covers one full year and, in fact, rates reached as high as 61% when taking into account only the transmission peak in Lupuna community. Second, differences in age distribution of malaria infections between the different locations. The proportion of gametocyte carriers is known to be higher among younger age groups in areas of high malaria transmission, where children have higher asexual densities [8]. Thus it is reasonable to expect an increase in gametocyte rates in a high transmission setting like PNG, where *P*.*vivax* infections concentrate in children <12 years old, as opposed to our study[15]. Third, factors attributable to different blood collection and parasite detection methods. On one hand, Barbosa *et al* processed 200μl of blood in Brazil, as compared to 50 μl blood collections both in the present (estimated volume) and in other studies [15,20,21]. Using high blood volumes can increase the chance to detect low-density gametocytemic samples; moreover, the few negative samples reported in that study had asexual parasite densities below 11 parasites/μl, levels that are highly frequent in our surveys. On the other hand, the performance of the all-stage parasite detection qPCR protocol may affect gametocyte rates by diminishing estimates when a highly sensitive asexual qPCR is used. Because gametocytes often represent only a small proportion of the total parasite biomass, the high rate of sub-microscopic infections found in this area compromises technical sensitivity for gametocyte detection, and may explain why gametocyte rates by RTqPCR were lower than those detected by LM. This effect was particularly relevant for *P*.*falciparum* (98% sub-microscopic rate), as the few sexual stage parasites detected were in either submicroscopic or asymptomatic infections. In fact, when only patent infections were taken into account, gametocyte rates by RTqPCR were close to 90%.

Sub-microscopic infections predominated in both communities independently of the observed differences in local transmission patterns. Overall, these results highlight the need of using highly sensitive molecular methods for malaria surveillance in pre-elimination settings. In this context, the relevance of sub-microscopic and asymptomatic infections (*i*.*e*. those that would escape routine LM-based diagnostics by field teams) resides largely on whether they contribute to effective mosquito infections and disease transmission. Although mosquito infectivity is known to increase with parasite density [37], *P*.*falciparum* infections at sub-microscopic levels have been suggested to account for as much as ≈30% of human-mosquito transmissions in countries like Burkina Faso [12,37,38]. For *P*.*vivax*, our understanding of the infectivity of sub-microscopic infections is much more limited, and the few data available has shown some variability, probably due in part to modest sample sizes [18,39]. A study in Brazil in which *Anopheles darlingi* fed directly on 11 *P*.*vivax* sub-microscopic patients found only 2 positive midguts [39], while Vallejo *et al* showed that 56% of naturally infected *P*.*vivax* sub-microscopic carriers from Colombia could infect *Anopheles Albimanus*, an infectivity rate similar to that of symptomatic carriers albeit with lower number of infected mosquitoes and oocyst counts [18]. Remarkably, the asymptomatic infections in the present study showed median gametocyte densities similar to those in symptomatic individuals. Moreover, a third of all gametocytemic patients were asymptomatic with patent parasitemia. Taken together, the data suggests that a lower success in infectivity of sub-microscopic infections might be compensated by the high frequency of both sub-microscopic and/or asymptomatic carriers (67% of *P*.*vivax* and all *P*.*falciparum* gametocyte carriers), as well as the relatively high *P*.*vivax* gametocyte densities in part of the asymptomatic population. In addition, asymptomatic parasite carriers will contribute to infectiousness for longer periods of time, as these individuals are not seeking treatment [40,41].

In terms of age groups, all those above 5 years of age were found to be more susceptible to both sexual and asexual *P*.*vivax* infections in this study, with a peak in >15–25 year old. This parallelism is in agreement with the strong association found between total parasite densities and presence of gametocytes. Conversely, other studies on *P*.*vivax* epidemiology have reported that prevalence of sexual stage infections decreases with age [10,15,19,20,23]. On one hand, individuals aged >15 years included here worked mainly in agriculture, forestry and fishing activities, what can increase their exposure to *Anopheles darlingi* bites during daytime. On the other hand, it is also reasonable to think that the sustained period of low transmission in Peru between 2005–2011 might have impacted immune patterns and shifted the acquisition of immunity towards older ages. This finding is in apparent contradiction with the observed high rates of sub-microscopic infections across all age groups, especially for *P*.*falciparum*. However, it has been suggested that after a period of reduction in malaria transmission, hosts might have better control of parasitemia and clinical disease provided that some immunity persists, time before reinfection is extended, and new infections are likely to be monoclonal [42]. Indeed, the study area experienced a reduction of the effective parasite population size due to a bottleneck event as a result of 2005–2011 control programs [43]. Whether age patterns will shift again after the increase in transmission observed since 2012 remains to be determined.

Significant differences in malaria indicators were found at community level, with residence in Lupuna being independently associated with higher infection rates in multivariate models. Differences between Cahuide and Lupuna communities are not surprising given their demographic and geographical characteristics. Whereas Lupuna is formed by forested riverine villages, Cahuide has scattered households, road-driven deforestation and a much more unstable population. Intense malaria control measures were applied in Cahuide in mid-2012 after a malaria outbreak, thus contributing to explain the lower infection prevalence. Furthermore, population genetic studies in the region showed that *P*.*vivax* population is highly structured suggesting different interactions with the host at the local scale [43]. The higher transmission in Lupuna was also accompanied by marked seasonality in *P*.*vivax* gametocyte carriage, which peaked one trimester before total infections did. However, since the addition of survey variable did not have a significant effect on the fit of multivariate model, differences in gametocyte rates between surveys are likely due to differences in total parasite densities rather than to a true seasonal effect. Overall, the detailed data obtained for two contrasting communities provides clues on what can be expected in other areas sharing similar characteristics and on how targeted interventions could be adapted.

This study has some limitations. On one hand, despite gametocyte carriage is a better indicator of the infectious reservoir than total parasite rates, it still remains indirect as compared to mosquito feedings, which have more control on factors like host and vector immunity. On the other hand, the trimestral sampling strategy allows for epidemiological characterization of gametocyte carriage, but does not allow for accurate calculation of the duration of gametocytemia; future studies on gametocyte dynamics in the area will contribute to answer these questions.

In conclusion, asymptomatic and sub-microscopic infections are significant contributors to the gametocyte reservoir in the Peruvian Amazon, despite the high degree of heterogeneity of transmission at the local scale and throughout the transmission season. Gametocyte prevalence peaks in young adults, but rates relative to asexual stage infections are similar across all age groups. Control and elimination campaigns need sensitive tools to detect infections that would otherwise escape routine malaria surveillance and may contribute to the maintenance of transmission in the Amazon region.

## Supporting information

S1 Checklist STROBE statement. (PDF)

S1 Table Characteristics of the study cohort at screening, by survey. (PDF)

S2 Table Univariate associations with *P. vivax* infections. Only significant results are shown (*P*<0.05).
(PDF)

S3 Table Multivariate models for *P. vivax* densities. (PDF)

S4 Table Univariate and multivariate associations with *P. falciparum* infections. For univariate analysis only significant results are shown (*P*<0.05). Association analysis was not attempted for sexual stage infections due to the low number of cases.
(PDF)

S1 Fig *Plasmodium* densities by qPCR/RTqPCR. A) *P*.*vivax and P*.*falciparum* parasite densities by qPCR as *18S* rRNA copy numbers/μl of blood. B) *P*.*vivax* and *P*.*falciparum* gametocyte density estimates as gametocytes/μl of blood by RTqPCR. Numbers in brackets indicate number of observations at each survey.
(PDF)
