# Supplementary material for: Predominance of asymptomatic and sub-microscopic infections characterizes the Plasmodium gametocyte reservoir in the Peruvian Amazon
Source: PLoS Negl Trop Dis. 2017 Jul 3;11(7):e0005674. doi: 10.1371/journal.pntd.0005674 (PMC5510906; doi:10.1371/journal.pntd.0005674)
Supplement: S1 Table — (PDF) [file pntd.0005674.s002.pdf]

S1 Table. Characteristics of the study cohort at screening, by survey.

	Dec-13		Mar-14		Jun-14		Sep-14		Dec-14		***P*-value**
	**n**	**%**	**n**	**%**	**n**	**%**	**n**	**%**	**n**	**%**	
Individuals sampled	1648	-	659	-	1653	-	1648	-	1657	-	**<0.001**
Age, *mean (95% CI)*	27.6 (4, 67)		26.1 (4, 66)		28.2 (4, 68)		28.0 (5, 68)		28.1 (5, 68)		0.190
Age											
≤5y	149	9.0	65	9.9	144	8.7	134	8.1	129	7.8	
>5y - 10y	260	15.8	112	17.0	260	15.7	279	16.9	279	16.8	
>10y - 15y	221	13.4	115	17.5	229	13.9	229	13.9	226	13.6	
>15y - 25y	269	16.3	86	13.1	246	14.9	248	15.0	260	15.7	
>25y	749	45.4	281	42.6	774	46.8	758	46.0	763	46.0	0.413
Male/female ratio	1.05	-	0.94	-	0.99	-	0.97	-	0.99	-	0.712
Work status											
Employed	551	33.5	198	30.1	554	33.6	537	32.6	538	32.5	
Students or children	736	44.7	322	49.0	730	44.2	745	45.3	750	45.3	
Other (including housework)	360	21.9	137	20.9	366	22.2	363	22.1	366	22.1	0.761
Households sampled	411	-	209	-	419	-	409	-	409	-	**<0.001**
House wall materials											
Brick, cement	39	9.5	19	9.1	37	8.8	36	8.8	35	8.6	
Wood	318	77.4	165	78.9	325	77.6	316	77.3	319	78.0	
Palm	36	8.8	15	7.2	37	8.8	37	9.0	35	8.6	
Other	18	4.4	10	4.8	20	4.8	20	4.9	20	4.9	0.990
Had malaria previous year	512	31.4	275	42.0	520	31.8	505	31.0	519	31.7	**0.001**
Visits with symptoms[a]	239	18.2	67	12.5	209	16.0	120	9.1	127	9.9	**0.001**
Clinical malaria cases[b]											
P. vivax	11	0.8	5	0.9	19	1.5	17	1.3	4	0.3	**0.030**
P. falciparum	1	0.1	0	0.0	0	0.0	1	0.1	0	0.0	0.667

[a]fever, headache and/or chills (among those with full clinical data available at the time of visit and during the previous 7 days); [b]by light microscopy at screening. CI, confidence interval.
