# Supplementary material for: Predominance of asymptomatic and sub-microscopic infections characterizes the Plasmodium gametocyte reservoir in the Peruvian Amazon
Source: PLoS Negl Trop Dis. 2017 Jul 3;11(7):e0005674. doi: 10.1371/journal.pntd.0005674 (PMC5510906; doi:10.1371/journal.pntd.0005674)
Supplement: S2 Table — Only significant results are shown (P<0.05). (PDF) [file pntd.0005674.s003.pdf]

S2 Table. Univariate associations with *P. vivax* infections. Only significant results are shown (P<0.05).

		**Parasite prevalence**		
**Variable**	**OR**	**95% CI**	***P*-value**[a]	
Age category				
≤5y	1			
>5y - 10y	2.04	1.18, 3.54	**0.010**	
>10y - 15y	2.55	1.46, 4,43	**0.001**	
>15y - 25y	3.35	1.95, 5.76	**<0.001**	
>25y	2.34	1.41, 3.88	**0.001**	***(<0.001)***
Village				
Cahuide	1			
Lupuna	2.07	1.62, 2.64	**<0.001**	
House wall				
Brick, cement	1			
Wood	1.36	0.84, 2.21	0.209	
Palm	1.73	0.93, 3.23	0.082	
Other	2.03	1.01, 4,10	**0.049**	*(0.168)*
Fever, headache or chills	2.24	1.72, 2.94	**<0.001**	

		**Gametocyte rate**		
**Variable**	**OR**	**95% CI**	***P*-value**[a]	
Village				
Cahuide	1			
Lupuna	1.62	1.08, 2.44	**0.019**	
Survey				
Dec-13	1			
Mar-14	1.05	0.50, 2.18	0.900	
Jun-14	0.97	0.55, 1.65	0.900	
Sep-14	0.46	0.26, 0.83	**0.010**	
Dec-14	0.64	0.35, 1.17	0.146	***(0.042)***
Asexual density (log)	2.16	1.88, 2.48	**<0.001**	
Fever, headache or chills	3.58	2.14, 5.99	**<0.001**	

		**Parasite density, by 18S**		
**Variable**	**Effect**[b]	**95% CI**	***P*-value**[a]	
Village				
Cahuide	1			
Lupuna	1.97	1.34, 2.94	**0.001**	
Work status				
Employed	1			
Students or children	1.99	1.28, 3.10	**0.002**	
Other	1.58	0.93, 2.69	0.086	***(0.007)***
Fever, headache or chills	5.75	3.60, 9.21	**<0.001**	

		**Gametocyte density**		
**Variable**	**Effect**[b]	**95% CI**	***P*-value**	
Asexual density (log)	1.14	1.01, 1.30	**0.048**	

[a]result of Wald test shown in brackets; [b]calculated as $e^{\beta \text{ regression coefficient}}$. OR, odds ratio; CI, confidence interval.
