# Supplementary material for: Predominance of asymptomatic and sub-microscopic infections characterizes the Plasmodium gametocyte reservoir in the Peruvian Amazon
Source: PLoS Negl Trop Dis. 2017 Jul 3;11(7):e0005674. doi: 10.1371/journal.pntd.0005674 (PMC5510906; doi:10.1371/journal.pntd.0005674)
Supplement: S3 Table — (PDF) [file pntd.0005674.s004.pdf]

S3 Table. Multivariate models for *P. vivax* densities.

	Parasite density, by 18S				Gametocyte density			
**Variable**	**Effect**[a]	**95% CI**	***P*-value**[b]		**Effect**[a]	**95% CI**	***P*-value**[b]	
Age								
≤5y	1				1			
>5y - 10y	1.46	0.49, 4.33	0.493		0.67	0.11, 3.90	0.657	
>10y - 15y	1.51	0.51, 4.51	0.457		0.63	0.11, 3.46	0.591	
>15y - 25y	1.39	0.48, 4.04	0.547		0.33	0.06, 1.73	0.188	
>25y	0.82	0.30, 2.27	0.704	*(0.109)*	1.05	0.21, 5.37	0.951	*(0.136)*
Village								
Cahuide	1				-	-	-	
Lupuna	1.59	1.05, 2.42	**0.028**		-	-	-	
18S copies/µl (log)	-	-	-		1.16	1.01, 1.32	**0.034**	
Fever, headache or chills	5.93	5.73, 9.17	**<0.001**		-	-	-	

[a]calculated as $e^{\beta \text{ regression coefficient}}$; [b]result of Wald test shown in brackets. CI, confidence interval.
