# Supplementary material for: Predominance of asymptomatic and sub-microscopic infections characterizes the Plasmodium gametocyte reservoir in the Peruvian Amazon
Source: PLoS Negl Trop Dis. 2017 Jul 3;11(7):e0005674. doi: 10.1371/journal.pntd.0005674 (PMC5510906; doi:10.1371/journal.pntd.0005674)
Supplement: S4 Table — For univariate analysis only significant results are shown (P<0.05). Association analysis was not attempted for sexual stage infections due to the low number of cases. (PDF) [file pntd.0005674.s005.pdf]

S4 Table. Univariate and multivariate associations with *P. falciparum* infections. For univariate analysis only significant results are shown (*P*<0.05). Association analysis was not attempted for sexual stage infections due to the low number of cases.

	Parasite prevalence (univariate)			
**Variable**	**OR**	**95% CI**	***P*-value**[a]	
Fever, headache or chills	0.31	0.25, 0.38	**<0.001**	
Survey				
Dec-13	1			
Mar-14	1.23	0.86, 1.77	0.256	
Jun-14	0.46	0.33, 0.65	**<0.001**	
Sep-14	0.14	0.09, 0.24	**<0.001**	
Dec-14	0.05	0.02, 0.11	**<0.001**	***(<0.001)***
	**Parasite prevalence (multivariate model)**			
**Variable**	**OR**	**95% CI**	***P*-value**[a]	
Age category				
≤5y	1			
>5y - 10y	0.70	0.33, 1.51	0.365	
>10y - 15y	0.89	0.41, 1.90	0.761	
>15y - 25y	0.36	0.15, 0.84	**0.018**	
>25y	0.59	0.30, 1.15	0.122	*(0.112)*
Fever, headache or chills	0.30	0.24, 0.37	**<0.001**	
Survey				
Dec-13	1			
Mar-14	1.65	1.03, 2.65	**0.037**	
Jun-14	0.50	0.32, 0.78	**0.003**	
Sep-14	0.20	0.10, 0.37	**<0.001**	
Dec-14	0.07	0.03, 0.18	**<0.001**	***(<0.001)***

[a]result of Wald test shown in brackets. OR, odds ratio ; CI, confidence interval.
