# Supplementary material for: Predominance of asymptomatic and sub-microscopic infections characterizes the Plasmodium gametocyte reservoir in the Peruvian Amazon
Source: PLoS Negl Trop Dis. 2017 Jul 3;11(7):e0005674. doi: 10.1371/journal.pntd.0005674 (PMC5510906; doi:10.1371/journal.pntd.0005674)

S1 Fig. *Plasmodium* densities by qPCR/RTqPCR. A) *P.vivax* and *P.falciparum* parasite densities by qPCR as 18S rRNA copy numbers/µl of blood. B) *P.vivax* and *P.falciparum* gametocyte density estimates as gametocytes/µl of blood by RTqPCR. Numbers in brackets indicate number of observations at each survey.

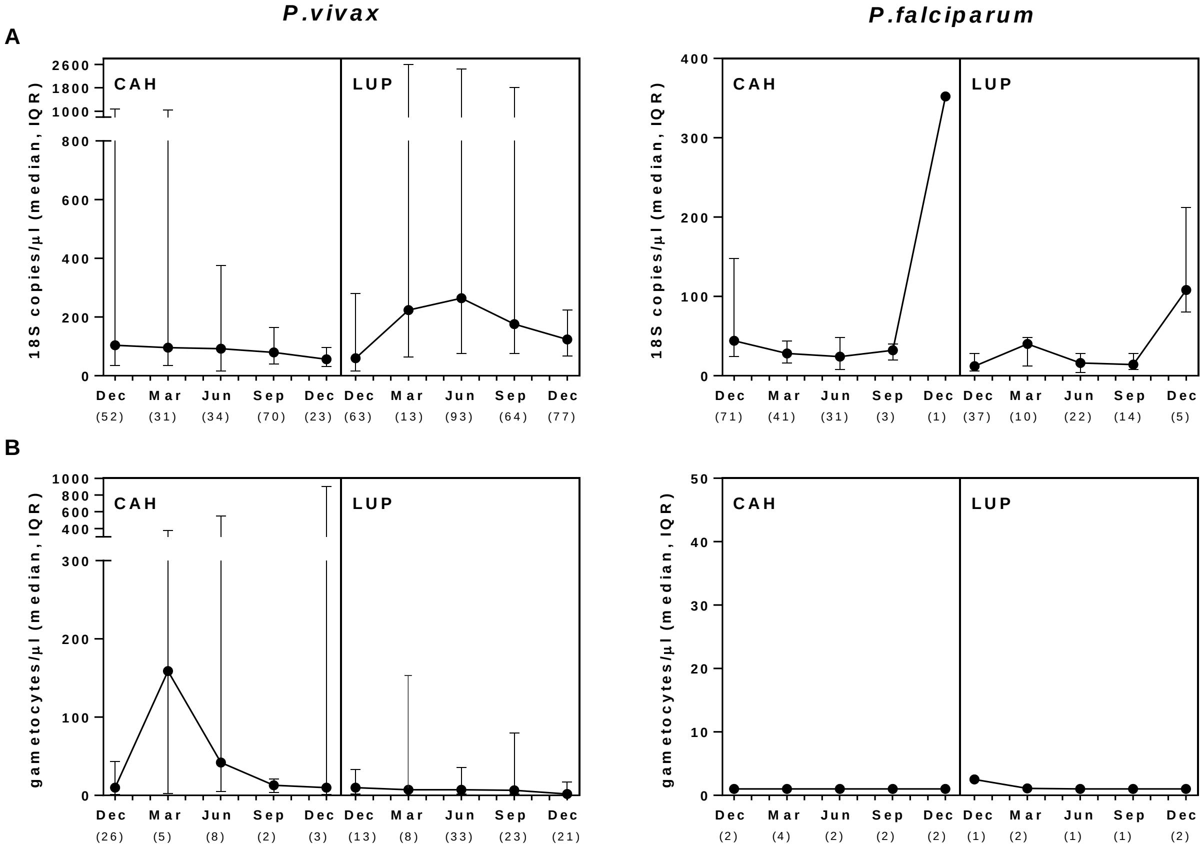

Supplement: S1 Fig — A) P.vivax and P.falciparum parasite densities by qPCR as 18S rRNA copy numbers/μl of blood. B) P.vivax and P.falciparum gametocyte density estimates as gametocytes/μl of blood by RTqPCR. Numbers in brackets indicate number of observations at each survey. (PDF) [file pntd.0005674.s006.pdf]
